# Higher Incidence of B Cell Malignancies in Primary Immunodeficiencies: A Combination of Intrinsic Genomic Instability and Exocytosis Defects at the Immunological Synapse

**DOI:** 10.3389/fimmu.2020.581119

**Published:** 2020-11-09

**Authors:** Jérôme Mastio, Mezida B. Saeed, Hannah Wurzer, Max Krecke, Lisa S. Westerberg, Clément Thomas

**Affiliations:** ^1^Department of Oncology, Cytoskeleton and Cancer Progression, Luxembourg Institute of Health, Luxembourg City, Luxembourg; ^2^Department of Microbiology, Tumor and Cell Biology, Karolinska Institutet, Stockholm, Sweden

**Keywords:** immunological synapse, actin cytoskeleton, membrane trafficking, exocytosis, cancer, primary immunodeficiencies, B cells, cytotoxic cells

## Abstract

Congenital defects of the immune system called primary immunodeficiency disorders (PID) describe a group of diseases characterized by a decrease, an absence, or a malfunction of at least one part of the immune system. As a result, PID patients are more prone to develop life-threatening complications, including cancer. PID currently include over 400 different disorders, however, the variety of PID-related cancers is narrow. We discuss here reasons for this clinical phenotype. Namely, PID can lead to cell intrinsic failure to control cell transformation, failure to activate tumor surveillance by cytotoxic cells or both. As the most frequent tumors seen among PID patients stem from faulty lymphocyte development leading to leukemia and lymphoma, we focus on the extensive genomic alterations needed to create the vast diversity of B and T lymphocytes with potential to recognize any pathogen and why defects in these processes lead to malignancies in the immunodeficient environment of PID patients. In the second part of the review, we discuss PID affecting tumor surveillance and especially membrane trafficking defects caused by altered exocytosis and regulation of the actin cytoskeleton. As an impairment of these membrane trafficking pathways often results in dysfunctional effector immune cells, tumor cell immune evasion is elevated in PID. By considering new anti-cancer treatment concepts, such as transfer of genetically engineered immune cells, restoration of anti-tumor immunity in PID patients could be an approach to complement standard therapies.

## Introduction

Primary immunodeficiency disorders (PID) represent a group of more than 400 inherited diseases that occur when at least one component of the immune system is either absent or dysfunctional (1, 2). PID patients have a higher risk of developing severe and often fatal infections, allergies, autoimmune complications, and malignancies (3, 4). Not long ago, these PID were considered rare conditions. However, with the advent of high throughput next generation sequencing technologies, the accuracy of PID diagnosis has markedly improved, especially for complex PID (5). Consequently, the incidence of PID has increased, from approximately 1/10000 to 1/1000–1/5000 during the last years (1). The discovery of new genetic defects continues to increase the incidence of PID. For these reasons, a better understanding of the PID related complications is critical to improve treatments and the clinical outcome of PID patients.

Among the complications associated with PID, cancer has received a particular attention. Several large cohort studies conducted in different parts of the world have shown an increased relative risk of cancer in PID patients (6–8). Cancer in PID often involves hematological malignancies that may stem from intrinsic failure to regulate genetic instability and cell transformation during hematopoietic cell differentiation and/or from altered tumor immunosurveillance by cytotoxic lymphocytes. During diversification of the B cell receptor (BCR) and T cell receptor (TCR), B and T cells undergo complex genetic alteration including DNA double strand breaks (DSB), DNA repair, cell proliferation and cell death (9). Tumor immunosurveillance involves multiple types of immune cells with cytotoxic lymphocytes, CD8^+^ T cells and Natural Killer (NK) cells, playing a major role in cancer cell elimination (10). Detection and eradication of cancer cells rely on the ability of cytotoxic lymphocytes to form a stable and functional lytic immunological synapse (IS). The IS can be defined as the interface between a cytotoxic immune cell and a target cell. The center of the IS consists of a closed nanoscale space termed synaptic cleft where most of the transcellular communications occurs. Both the cytotoxic cell and the target cell engage multiple complementary receptors and ligands across the synaptic cleft. For CD8^+^ T cells, the interaction between the T cell receptor (TCR) and the class I major histocompatibility complex (MHC) is critical for killing of the target cell. For NK cells, the recognition of activating and inhibitory ligands by NK cell specific receptors drives the killing response. These interactions trigger intracellular signals that allow cytotoxic lymphocytes to respond to the encountered cell accordingly. When the cognate target cell is a healthy cell, an inhibitory synapse is formed. This inhibitory synapse prevents the engagement of the cytotoxic lymphocytes lytic machinery and thus preserves healthy cells from cytotoxic deleterious effects (11). When cytotoxic cells are in contact with antigen presenting cells (APC), such as dendritic cells (DC), the lytic cascade is prevented and the synapse that is assembled is called a regulatory synapse (11). Regulatory synapses are required to promote CD8^+^ T cell priming by DC, but also DC licensing by CD4^+^ T cells. Finally, if the target cell is recognized as malignant, a lytic IS is formed (11). A characteristic of the lytic IS is the secretion of cytotoxic molecules within the cleft. Once released, the cytotoxic molecules penetrate the cancer cell and induce cell death, mostly by apoptosis. Directed release of cytotoxic granules toward the target cell guarantees that surrounding healthy cells are not affected by the immune surveillance defense mechanism (12). In PID patients with aberrantly functional cytotoxic lymphocytes, the immune surveillance is impaired (1, 2). As a result, the appearance and progression of malignancies are common in PID patients. Dysfunctions of cytotoxic cells can occur at several stages during cancer cell killing and can affect multiple subcellular networks. For instance, the IS is frequently altered in PID patients. Most of the time, the loss of molecules in the actin and microtubule cytoskeleton causes defective IS formation. The actin and microtubule cytoskeleton are needed to support the architecture and dynamics of the IS, notably the three concentric supra-molecular activation clusters (SMACs) (central, peripheral and distal) observed at the T cell IS (13). In addition to mutations in cytoskeletal proteins, mutations in molecules of the exocytosis machinery are commonly found among PID patients.

In this review, we will first address the different factors that increase the risk of cancer in PID patients. We will specifically focus on the most represented cancer types found in the PID population, namely lymphomas and leukemia and why they develop. We will thereafter present genetic defects classified as PID and known to impair either the formation, stability or function of the IS. Any alterations affecting regulatory synapses will also be discussed (11).

## Malignancies in PID Patients

### PID Associated Malignancies

Over the past decade, advances, and availability of genetic diagnostic tools such as next generation sequencing has shed light on the prevalence of PID with ≈ 400 disorders identified (2). Large multi-centre studies in different countries have also been conducted to describe the complexity of clinical presentations (14). Systematic reviews of epidemiological data describing the frequency and nature of malignancies associated with PID has yielded surprising insights. Firstly, malignancies occur more frequently and earlier in life in PID patients (15). Secondly, when compared to cancer incidence in general, malignancies associated with PID are narrower in range with higher incidence of hematological malignancies, with non-Hodgkin lymphomas, diffuse large B cell lymphomas, marginal zone lymphomas and Burkitt lymphomas accounting for a high proportion of these disorders (16). Finally, the nature of PID associated malignancies is different from malignancies caused by secondary immunosuppression. In transplant recipients or patients with acquired immunodeficiency syndrome most malignancies present as melanoma and renal cell carcinoma. However, melanoma and renal cancer carcinoma is uncommon in PID patients (17). Taken together, these observations argue for an intrinsic role of the PID causing genes in molecular mechanisms of genomic stability and cell transformation. The development of antigen specific immunity requires the generation of B and T cells bearing antigen specific receptors that are expressed upon complex rearrangements of gene segments followed by cell proliferation and cell death during selection of antigen receptor bearing B and T cells. This remarkable system enables the host to develop an astounding diversity in adaptive immune cells against a wide range of pathogens. It is not surprising that the large network of molecular players involved in the genetic rearrangements, proliferation and apoptosis make B and T cells highly susceptible to cellular transformation. Here, we focus on the development of B cells to first describe the molecular machinery required for generating receptor diversity and then discuss aspects of this process that predispose to PID with malignancies.

### Programmed DNA Repair and Proliferation of B Cells

#### V(D)J Recombination

**Figure 1:**
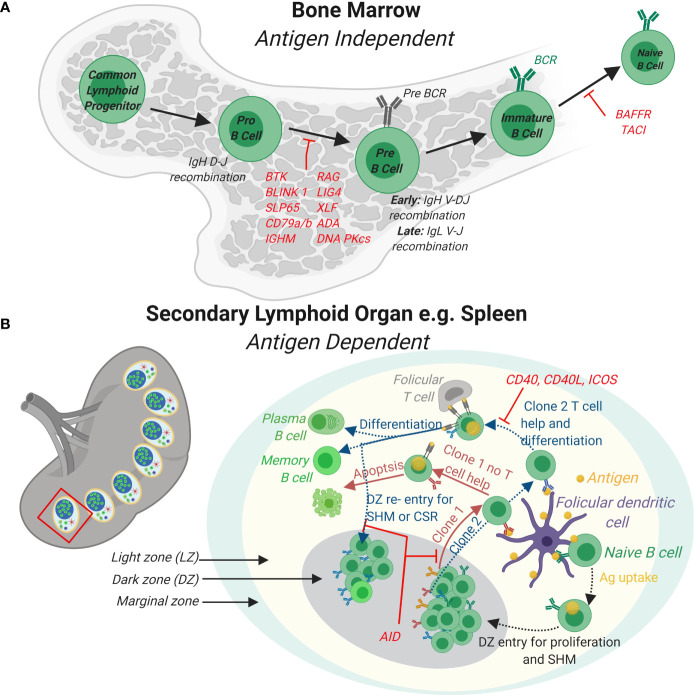
B cell development in the bone marrow and periphery. **(A)** In the bone marrow, B cells develop from Common Lymphoid Progenitor cells to naïve B cells expressing a functional B cell receptor (BCR) in a series of developmental steps. Firstly, expression of lymphocyte specific RAG complexes triggers Pro-B cell development by initiating the recombination of the D and J segments of the Immunoglobulin heavy chain (IgH) and expression of surrogate light chains. Further differentiation of Pre-B cells leads to the rearrangement of the complete IgH and also Ig light (IgL) gene. Immature B cells with functional BCRs receive stimulatory signals completing naïve B cell development in the bone marrow. **(B)** In response to antigens, naïve B cells develop further into effector B cells in peripheral lymphoid organs such as the spleen (shown) or lymph nodes (not shown). In the spleen, germinal centers (GCs) that are specialized structures that contain follicular dendritic cells and T follicular helper cells are formed. A GC contains a light zone (LZ) and a dark zone (DZ). The marginal zone surrounds the GC. Naïve B cells take up antigen presented by follicular dendritic cells in the LZ. BCR signaling induces B cell proliferation in the DZ and triggers AID somatic hyper mutation (SHM) of the BCR. B cells expressing newly formed clones (e.g. clones 1 and 2) re-enter the LZ to take up antigen from follicular DCs and present these *via* major histocompatibility complex (MHC) II to T follicular helper cells. Clones (e.g. clone 1) that do not receive T cell help *via* CD40-CD40 L signaling axis fail to differentiate and survive. Clones that receive T cell help (e.g. clone 2) differentiate to antibody secreting plasma cells or long-lived memory cell or re-enter the DZ for further SHM and class switch recombination (CSR). Loss of function mutations for genes involved and that cause PID are indicated in red.

**Figure 2:**
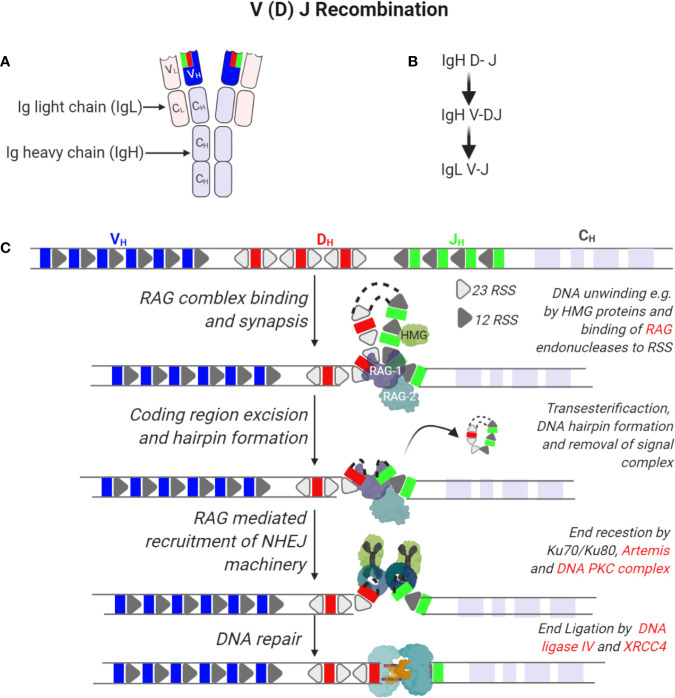
Recombination of immunoglobulin V (variable), D (diversity), and J (joining) segments of immunoglobulin heavy and light chains. **(A)** Tertiary structure of an antibody indicating two molecules of immunoglobulin heavy (IgH) and light chains (IgL). Each IgH is composed of a variable region (VH) derived from the V(D)J segments of the IgH gene and constant (CH) regions. On the other hand, IgL is composed of a variable region (VL) derived from recombining V and J segments of the IgL gene and smaller constant regions (CL). **(B)** Sequence of immunoglobin gene segment recombination. **(C)** The IgH gene is composed of V, D, and J gene segments that are flanked by recombination signal sequences (RSS) that are 23 or 12 nucleotides long. In the first step of IgH recombination, D and J segments access is enabled by unwinding of DNA in these regions by e.g. HMG proteins enabling the binding of a complex of recombination-activating genes 1 and 2 (RAG1/2) randomly to any of these segments. Endonuclease activity by RAG1/2 enables removal of intervening sequences, formation of DNA hairpins and recruitment of the non-homologous end joining DNA repair machinery. Here, hairpins are opened by the activity of Ku70/80, Artemis and DNA PKC complexes while ends are ligated by the activity of XRCC4 and DNA ligase IV.

B cells are characterized by the expression of the BCR that is composed of the immunoglobulin (Ig) heavy (IgH) and light (Igκ or Igλ, collectively IgL) chains (18). Antibody producing B cells in the periphery develop from common progenitor cells in a series of developmental processes in the bone marrow and in secondary lymphoid organs such as lymph nodes and spleen (**Figure 1**) (19). The genomic rearrangements to generate the BCR serve to increase the repertoire of antigens that can be identified by the BCR (via V(D)J recombination and somatic hypermutations, SHM) as well as the efficacy of the antibody response (Ig class switch recombination, CSR). Development in the bone marrow results in the formation of naïve B cells that express a functional BCR that contain a unique combination of IgH and IgL chains. The IgH is the product of joining germline V (variable), D (diversity) and J (joining) gene segments while the IgL is a product of recombining V and J gene segments only (20). Both rearrangements are mediated by the lymphocyte-specific recombination activation gene 1 (RAG1) and RAG2 endonucleases (21–23). The RAG1/2 complex initiates the Ig loci rearrangement by introducing DNA DSB through binding to recombination signature sequences (RSS) that flank the V(D)J segments and introduces a nick that exposes a 3’-OH group to form a hairpin by a transesterification reaction (**Figure 2**) (24, 25). The hairpin structures can be resolved in different ways to generate palindromic P elements. Exonucleases can remove nucleotides from the ends of the rearranged genes. The enzyme terminal deoxynucleotidyl transferase (TdT) randomly adds non-germline-encoded N nucleotides to the ends of the rearranged genes before they are joined. The RAG1/2 mediated DSB are repaired by the classical non-homologous end joining (NHEJ) repair machinery (9). During NHEJ, the Ku70 and Ku80 end-binding complex recognizes DSB and the XRCC4 and DNA Ligase 4 complex joins them (9). Ku70/Ku80 recruits the DNA dependent protein kinase catalytic subunit (DNA-PKc) that activates end processing by the Artemis endonuclease (26, 27). Several DNA polymerases may contribute to end polishing including Polη (28). DNA DSB activate the ATM-dependent DNA damage response (DDR) in which ATM phosphorylates numerous substrates that mediate cell-cycle checkpoints and DNA repair, including p53 for cell cycle arrest and the chromatin-associated proteins H2AX and 53BP1 that binds to DSB (29). In contrast to homologous recombination (HR) that depends on large stretches of homologous sequence to guide repair and takes place in the S phase of the cell cycle, NHEJ repairs DNA breaks throughout the cell cycle using a spectrum of ends. These include DNA breaks lacking homology (direct joining) to those employing short microhomology sequences often from single strand overhangs from the break to guide repair (9). During the repair process, additional nucleotides are incorporated into the junctions from P and N nucleotides. The nucleotide changes caused by NHEJ repair, most often additions, is termed junctional diversity because of the large contribution to the diversity of BCRs that can be produced. An important mechanism to ascertain that one B cell expresses only one specific combination of IgH and IgL chains is allelic exclusion. Upon productive rearrangement of the IgH and IgL chains, successful signaling by the surface expressed BCR silences rearrangement of the additional alleles encoding the IgH and IgL chains. Allelic exclusion was discovered based on allotypic markers for each of the IgH alleles to distinguish surface expression of the two IgH alleles (30).

#### Somatic Hypermutation and Germinal Center Response

**Figure 3:**
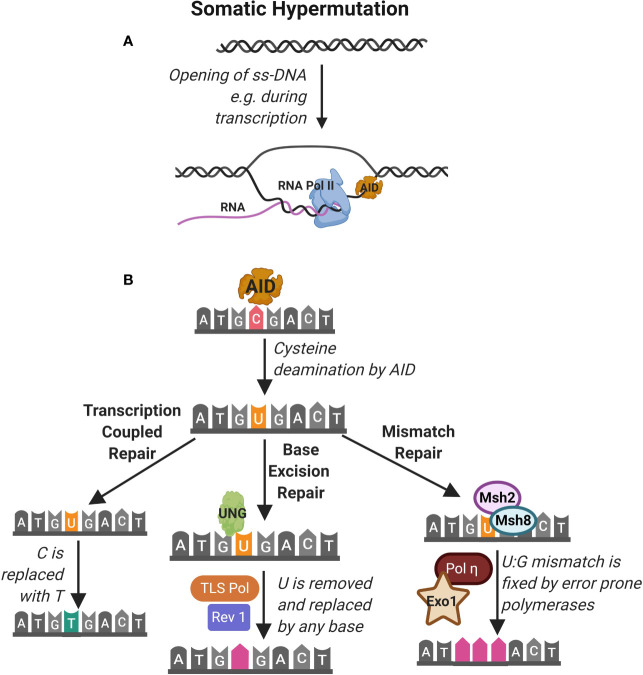
Somatic hypermutation. **(A)** Somatic hypermutation of the immunoglobulin heavy chain is mediated by the activity of the enzyme activation-induced cytidine deaminase (AID). AID accesses nucleotides on single strand DNA (ss-DNA) during transcription of RNA e.g. by RNA polymerase II (RNA pol II). **(B)** AID deaminates cytidine (C) nucleotides converting them to uracil (U). This is identified as DNA damage that can be repaired by (1) transcription coupled repair where the uracil is transcribed as thymidine (T), (2) base excision repair (BER) pathway where Uracil-DNA glycosylase (UNG) detects and excises U leading to replacement by any base by the TLS polymerase and Rev1 and (3) mismatch repair (MMR) pathway where Msh2 and Msh8 identify the U and repair mismatch using error prone Pol and Exo1.

During an immune response, transient structures called germinal centers (GC) form in secondary lymphoid organs such as the spleen and lymph nodes. In GCs, B cells undergo further maturation to become memory cells and plasma cells that produce high-affinity antibodies (**Figure 1**) (31). Affinity maturation describes the evolution of high affinity antibody producing B cells in GCs through iterative rounds of somatic hypermutations (SHM) in the variable region of the BCR and clonal expansion (32). SHM is mediated in GCs through the activity of the enzyme activation-induced cytidine deaminase (AID) (33–35). AID belongs to the APOBEC family of RNA editing enzymes and catalyzes deamination of cytidine *in vitro*, from deoxycytidine (dC) into deoxyuridine (dU) in V segments of the IgH and IgL genes (**Figure 3**) (35). Uridine nucleotides in DNA are recognized and removed by the Uracil-DNA glycosylase (UNG) (36). UNG triggers DNA repair *via* multiple pathways including the base excision repair (BER) or mismatch repair (MMR) pathways. These DNA repair pathways are error prone and introduce nucleotide exchange and additional nucleotide mutations during the repair of the V segment, diversifying the BCR. Affinity maturation through SHM is important for development of the robust immune response to pathogens, as highlighted in the many PID that affect the GC response leading to aberrant B cell antibody responses (37). For instance, patients lacking AID or UNG activity and therefore SHM, develop hyper IgM syndrome and are prone to severe bacterial infections (38). Interestingly, SHM in GCs upon an infection may be important to prevent expansion of autoreactive B cells since a vast majority of germline encoded antibodies produced by early B cell precursors contain autoreactive epitopes (39, 40).

GC B cells that have undergone SHM of the BCR are subjected to fierce competition for survival and clonal expansion. Competition involves BCR mediated binding of native antigen presented on follicular dendritic cells (FDC) and the availability of limited help from T follicular helper (Tfh) cells (41). GCs contain dark and light zone areas (DZ and LZ, respectively). Pulse chase experiments and more recently multiphoton intravital imaging studies revealed that follicular B cells are highly dynamic and migrate considerably between the LZ and DZ (42, 43). Moreover, GC B cells form short dynamic interactions with antigen presenting FDC and Tfh cells in the LZ while the DZ is filled with proliferating B cells expressing AID. Thus, B cells form specialized intracellular contact sites to capture antigens from the FDC and to present antigen on MHC class II molecules to receive help *via* IS formation with Tfh cells (44). B cells that have the highest affinity receptor for the antigen will capture the highest quantity of antigens, leading to increased antigen presentation to Tfh cells and thereby receiving an increased help from Tfh cells to initiate the memory cell and plasma cell program (44, 45). Therefore, synaptic interactions *via* IS are critical for the expansion, contraction and selection of GC clones as they provide important feedback on the strength of BCR signaling and efficiency of antigen presentation. B cells interactions with Tfh cells are critical in GC formation and propagation as (1) inborn errors in the CD40 receptor expressed on GC B cells or the CD40 ligand (CD40L) expressed on Tfh cells cause PID with complete lack of GCs and (2) CD40/CD40L blocking antibodies are sufficient to stop an ongoing GC reaction (46, 47).

#### Ig Class Switch Recombination

**Figure 4:**
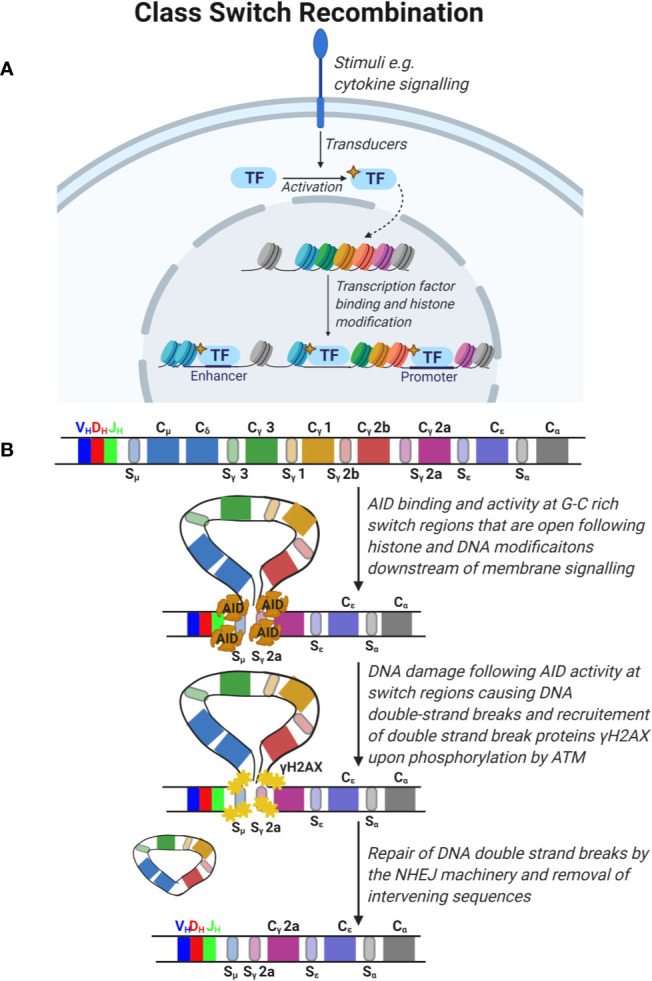
Class switch recombination. **(A)** Signals from the microenvironment *via* cytokine signaling are transduced in B cells to activate specific transcription factors which translocate to the nucleus and promote the opening of condensed DNA regions at specific areas and expose the switch regions 5’ of the immunoglobulin constant (C) segments. **(B)** The constant region of the immunoglobulin gene is composed of constant segments that encode different antibody subclasses. Constant regions are flanked by G-C rich switch regions. Deaminase activity of activation-induced cytidine deaminase (AID) leads to DNA damage at these switch regions triggering the double strand break repair response by ATM which phosphorylates H2AX and recruitment of the non-homologous end joining (NHEJ) machinery. This leads to DNA double strand break repair and removal of intervening sequences.

An important aspect of adaptive immunity is the functional diversification of antibodies from IgM and IgD subclasses, co-expressed on immature B cells, to IgG, IgA, and IgE antibody subclasses encoded by the constant (C) segments (Cµ, Cδ, Cγ, Cα, Cε) in the IgH chain (**Figure 4**). The functional antibody isotype is determined by the microenvironment where B cells integrate signals from cytokines and receptor interactions to produce different antibody isotypes. This process is known as Ig CSR and involves the introduction of double strand breaks in the switch regions 5´ of each IgH constant region (**Figure 4**) (48). Similar to SHM, CSR is mediated by the activity of AID. AID deaminates cytidine to uracil in the switch regions in a process that involves formation of DNA-RNA R-loops with non-template single stranded DNA. Due to the high frequency of GC nucleotides in the switch regions, AID deamination and UNG removal of uracils lead to double strand breaks that are repaired by the error prone BER and MMR pathways. The repair results in short (1-4 bp) addition of nucleotides, termed microhomology regions, between the joined switch regions. Chromatin remodeling and loop extrusion are critical features of CSR for ligation of double strand breaks in the two switch regions (**Figure 4**) (49). Many malignancies result from faulty repair of DNA double strand breaks during CSR, which can lead to c-myc and Bcl6 oncogenes translocations to the IgH chain locus under the control of the powerful promoter normally required for BCR expression (29).

### Malignant DNA Repair and Proliferation in B Cells

A hallmark of cancer is genomic instability leading to cellular transformation. The multiple rounds of DNA damage and repair required for B cell development makes B cells susceptible to the absence of molecules involved in repair pathways and PID can arise from the functional loss of such genes. B cell lymphomas comprises a heterogeneous group of malignancies that can be distinguished using clinical and pathological methods owing to subtype resemblance to B cells at a stage of differentiation as well as to gene expression profiling (50). Genetic studies have identified chromosome translocations as the most frequent genetic lesions in B cell malignancies (51). These can include deregulated expression by the juxtaposition of promoters or enhancers from other chromosomes such as translocation of c-myc under the enhancer of the IgH locus or fusion of genes on two separate chromosomes such as translocation leading to chronic myeloid leukemia (CML) and B cell acute lymphocytic leukemia (B-ALL). These translocations occur because of aberrant repair of DSBs (52). DSB are obligate intermediates during V(D)J recombination and as such most leukemia and lymphomas are associated with aberrant RAG activity at the Ig locus. Moreover, DSBs are also obligate intermediates during CSR and the occurrence of breakpoints around switch regions implicates AID activity. A frequent trigger of B cell lymphomagenesis are viral infections such as Epstein-Barr virus (EBV) that among other effects induces AID expression (53). For PID, the underlying etiology for the development of lymphomas are DNA repair defects and EBV associations (16). The treatment of PID patients is similar to other lymphomas patients with some form of combination chemotherapy, most commonly, rituximab, cyclophosphamide, doxorubicin, vincristine and prednisolone (R-CHOP). However, the mortality rate is higher for PID patients (16). In most cases, as in other cancers, genomic instability itself is not sufficient to induce cellular transformation. B cell malignancies often commandeer and misuse normal signaling and regulatory pathways to sustain their growth and survival including constitutive activation of the anti-apoptotic NK-κB signaling (50). PID with mutation in repair proteins, such as DNA ligase 4, Artemis (54), XRCC4 (55), XLF/Cerunnos (56, 57) have reduced or lack NHEJ and may use an alternative end joining (A-EJ) mechanism that give rise to longer microhomology regions (> 4 bp) (58). Naturally, many of the repair enzyme-mediated PID are associated with B cell deficiency, radiosensitivity and increased risk to develop malignancies, including but not limited to hematological malignancies (29). Deficiencies in any of the DNA damage response pathway proteins ATM (Ataxia–telangiectasia) (59), H2AX (28), or 53BP1 (60) in B cells activated for CSR lead to high levels of AID-dependent IgH chromosome breaks and translocations, similar to deficiencies in NHEJ.

PID with genetic mutations in proteins required for B cell development show complete loss of B cell lineage cells such as loss-of-function mutation in Bruton tyrosine kinase (Btk) (61), Igα (CD79a) (62) and RAG1/2 (63) (**Figure 1**). Other PID have leaky B cell development with alterations of the B cell diversity autoimmunity and B cell malignancies, jointly termed common variable immunodeficiency (CVID) and include PID with mutations in BAFF and the BAFF receptor TACI (64), BLNK/SLP65 (mouse) (65) and ICOS (expressed on Tfh cells) (66) (**Figure 1**). Many PID lead to autoantibody formation mainly caused by altered B cell maturation and selection in GCs. These include PID with mutations in actin regulators, dedicator of cytokinesis 8 (DOCK8), WASp interacting protein (WIP), Wiskott-Aldrich syndrome protein (WASp) and actin-related protein 2/3 (Arp2/3) (67). As in other diseases with autoantibodies, these PID patients are at risk of developing B cell malignancies although the underlying mechanism for B cell transformation has often not been identified (16). Interestingly, for AID deficiency, the abnormally large GCs seen in both patients and mouse models do not develop into malignant lymphomas, perhaps due to the lack of the AID induced DNA damage response (33, 34, 38, 68, 69).

### An Emerging Role for Actin Regulators in Genomic Stability and Cell Transformation

It is not surprising that genetic mutations affecting the expression of proteins involved in the DNA repair machinery are associated with cell transformation. It is also clear that malignancies, especially lymphoma, and many other PID, are associated with EBV infection. An interesting new concept is that mutations in regulators of the actin cytoskeleton may lead to cell intrinsic aberrations in maintenance of genetic stability and suppression of transformed cells. Regulation of actin dynamics has been implicated in cellular processes such as cytokinesis and genomic stability that are often dysregulated in malignant transformation (70). Specifically, most of the PID causative actin regulators, including Coronin-1A (71, 72), Rac2 (73), Dock2 (74), Dock8 (75), Moesin (73), RhoH (76), WIP (77), ARHGEF1 (78), CARMIL2 (79), and MKL1 (80), have been associated with increased predisposition to malignancy in multicenter or case studies, animal models or identified as specific biomarkers for specific malignant transformations.

Actin dynamics in immune cells are critical for cellular activation and function. To what extent the actin cytoskeleton intrinsically regulates genomic stability in addition to its well described cytoplasmic role in immune cell activation are only now beginning to be parsed out. This complex interplay and the challenge in untangling the two can be illustrated with the hematopoietic linage restricted actin regulator Wiskott-Aldrich syndrome protein (WASp). WASp is perhaps the best studied actin regulator and WASp associated-PID include both loss and gain of function [causing Wiskott-Aldrich syndrome (WAS) (81) and X-linked neutropenia (XLN), respectively (82–84)]. These mutations are associated with aberrant functioning of multiple types of immune cells including Natural Killer (NK) cells (85–87), B cells (88–94), and T cells (95–97). Both gain of function and loss of function mutations in WASp have been associated with higher risk of malignancies. The tumor incidence in WAS is estimated to be 13%–22% with a median age of onset of 9.5 years and with poor prognosis (98, 99) and most frequently lymphoreticular tumors including non-Hodgkin lymphoma (76% of the total tumors associated with WAS), Hodgkin lymphoma, and Burkitt lymphoma (99–105). Both reduced tumor immunosurveillance by cytotoxic cells (85–87, 106, 107) and intrinsic cell transformation (107–109) contribute to malignancies in WAS. In the first XLN family with constitutively active WASp (WAS-L270P) (82), two out of six affected males have developed myelodysplastic syndrome and leukemia and one unrelated XLN patient with the WAS-I294T mutation developed myelodysplastic syndrome (83). Interestingly, somatic loss-of-function and gain-of-functions of WASp have been described in patients with lymphomas and juvenile leukemia (110, 111). Here, we discuss how WASp that has a key role in synchronizing antigen receptor signaling with actin cytoskeleton dynamics (112, 113) could also play a role in preventing cell transformation.

#### Cytokinesis

Actin is critical during cell division to correctly segregate chromosomes and during cytokinesis by regulating the contractile ring of actin and myosin that cleaves the cell into two daughter cells (114, 115). During cell division, the actin cytoskeleton in adherent cells undergoes well characterized morphological changes enabling cells to detach from the substratum, round up and increase cortical rigidity, divide, and is reassembled after cytokinesis. These changes are mediated by coordination of cell cycle progression signals and the actin cytoskeleton (116). Increased actin polymerization during mitosis is associated with genomic instability and transformation. Increased activity of WASp in XLN patients leads to increased polymerized actin that during mitosis abnormally localizes around the mitotic spindle and chromosomes throughout their alignment and separation, and also accumulates within the cleavage furrow around the spindle midzone (117). This results in genomic instability as evident in polyploid cells and cells with micronuclei and aberrant chromosomes (117–119). These abnormalities were attributable to increased actin filament content in the cells accumulating around mitotic chromosomes and delaying mitosis. Moreover, increased accumulation of polymerized actin in the cleavage furrow impeded cytokinesis. Importantly, this defect was a mechanical impedance caused by the increased actin content of the cells as these defects could be induced in WASp sufficient cells by treatment with Jasplakolide that stabilizes actin filaments or rescued by inhibiting actin polymerization *via* the Arp2/3 complex using the small molecule inhibitor CK666 (117, 118). B cells expressing constitutively active WASp show decreased Ig CSR, cell proliferation, and increased apoptosis associated with genetic instability (119).

#### Genomic Stability

A phenotype observed in WAS T cells is the reduced inability to generate type 1 helper cells (120). One mechanism for this could be a reduced efficiency of IS formation by WAS T cells (121–123). Alternatively, WASp could play a role in genetic regulation of T cells. In this regard, chromatin immunoprecipitation (ChIP) experiments have revealed an association of WASp with TbxT1 genes (124, 125). Moreover, RNA sequencing analysis revealed different genetic regulation in the presence and absence of WASp (126). As WASp lacks DNA binding motifs, it is possible that these effects are in part mediated *via* its actin polymerization activity. The role of actin filaments in nuclear biology has been a matter of controversy for much of its history. While a case could be made for the presence of actin monomers in the nucleus due to the presence of specific nuclear import and export components, visualizing actin filaments, or conceptualizing biochemical signals that would drive actin polymerization has been technically challenging (127). Emergence of tools to specifically study actin filaments in the nucleus using specific reporters with nuclear localization signals as well as the presence of filament regulators such as WASp and Arp2/3 in the nucleus have reignited interest in this field. A role for nuclear actin filaments has been described in (1) homologous repair of heterochromatic double strand breaks in Drosophila oocyte where actin nucleation *via* Arp2/3 generate tracks for myosins to transport heterochromatic double-strand break to the nuclear lamina (128, 129), (2) regulating gene expression following serum starvation *via* N-WASp/RNA polymerase II interactions (130) and (3) in regulating the expression of key cytokines downstream of T cell receptor engagement (126, 131).

#### Mechanosensing in the Nucleus

Nuclear actin polymerization is linked to functional integrin signaling at the cell surface and components of the Linker of Nucleoskeleton and Cytoskeleton complex (LINC), suggesting that cellular adhesion and mechanosensing between the plasma membrane and nucleus contribute to nuclear actin dynamics (132). Aurora A and Aurora B jointly coordinate chromosome segregation and anaphase microtubule dynamics (133). Aurora A regulates microtubule dynamics and Aurora B regulates chromosome alignment at metaphase and delays abscission in cells with structural defects or chromatin bridges (134). Elevated F-actin caused by increased WASp activity such as in XLN patients leads to increased cell viscosity and activation of the Aurora B during mitosis (118). Aurora B activity is crucial in sensing mechanical changes to the centromere during chromosome alignment (134). Aurora B is interesting since it is a target for hematopoietic malignancies. A selective Aurora B inhibitor induces growth arrest and apoptosis by the accumulation of 4N and 8N DNA content of human acute leukemia cells *in vitro* and *in vivo* (135). Overexpression of Aurora B can prevent polyploidy in cells that have chemically and genetically induced mitosis defects (136) and rescue mitosis defect in cells expressing constitutively active WASp (118). The megakaryoblastic leukemia 1 (MKL1) is an actin sensor regulated by integrin signaling that binds globular (G)-actin in the cytoplasm. When the cytoplasmic G-actin concentration drops, MKL1 shuttles into the nucleus where MKL1 interacts with the serum response factor (SRF) to regulate expression of genes involved in the actin cytoskeleton. MKL1 deficiency results in severe immunodeficiency diseases (137). Increased MKL1 expression is associated with increased actin content in patient B cells leading to increased genomic instability and development of Hodgkin lymphomas (138). Together, this data suggests that increased actin content is associated with genetic instability and malignancies. Our understanding of the nuclear actin contribution in PID caused by mutations in actin regulators is in its infancy and may reveal unexpected causes for cell transformation and development of malignancies in PID.

## Genetic Defects Affecting the Formation, Stability, and Function of the IS

Multiple intracellular changes occur during immune cell activation upon target cell recognition, especially in regard to the actin and microtubule cytoskeletons. Together with the exocytosis machinery, these cytoskeletons regulate the adhesion to the target cell, the activation of the immune cell and the release of cytotoxic granules at the IS. Any alterations in these processes result in suboptimal eradication of cancer cells. PID with such defects are discussed in this second part. The first section is dedicated to issues mostly affecting the formation and the stability of the IS, along with their consequences on immune cells activation. PID with altered exocytosis of cytotoxic granules are addressed in the second section.

### PID Affecting the Formation and the Stability of the IS

**Figure 5:**
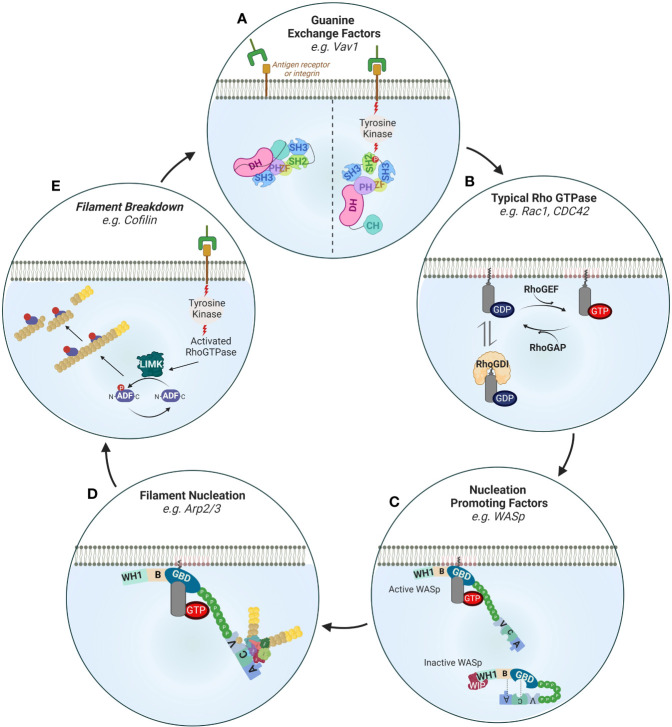
Regulation of actin filament turnover. **(A)** Guanine exchange factors such as Vav1 are activated downstream of receptor tyrosine kinase (RTK) signaling. Upon phosphorylation of its SH2 domain, Vav1 undergoes intramolecular reorganization that releases its nucleotide exchanging Dbl homology (DH)-pleckstrin homology (PH). Other important GEFs in immune cells are the DOCK family members (not shown). DOCKs are structurally different from the Vav family members in important ways. Namely, they contain lipid-binding domains (and thus are directly recruited to membranes) and lack DH-PH domains requiring association with the Elmo family of proteins to exchange nucleotides. **(B)** The activity of typical Rho GTPases is regulated by 1) GTP cycling and 2) intracellular retention by Rho GDI proteins. Rho GEFs such as Vav1, DOCK2 or DOCK8 enhance the exchange of GDP to GTP while Rho GAPs switch off Rho GTPases by enhancing the exchange of GTP with GDP. An important mechanism of signaling specificity is the preference of certain GEFs for certain Rho GTPases e.g. DOCK-2 to Rac, Gef-1 to CDC42 (not shown). **(C)** Nucleation promoting factors such as WASp are activated by Rho GTPases by relieving intramolecular folding (shown) or as in the case of the NPF WAVE regulatory complex by stabilizing a specific orientation (not shown). Inactive WASp in the cytoplasm is autoinhibited through interactions of its Basic Region (BR) with the Acidic (A) domain as well as its GTPase binding domain (GBD) with the central (C) domain. Interaction with activated CDC42 (through the GBD) and recruitment to the plasma membrane (through PIP2) relieves this inhibited conformation. Through its WH1 domain WASp also interacts with WIP which regulates its activity. **(D)** Activated membrane-proximal WASp interacts with the Arp2/3 complex to mediate the nucleation of new filaments at a 70° angle by direct binding of its CA domains to Arp2/3 and to profilin and actin through its verprolin (V) domain. Other important actin nucleators are the formins that are also similarly activated by relief of autoinhibitory intramolecular folding following interaction with Rho GTPases downstream of tyrosine kinase signaling (not shown). **(E)** Receptor tyrosine kinases also activate filament breakdown which is important in maintaining a pool of profilin actin monomers that can be used for *de novo* actin polymerization as well as feedback regulation of activation. A key protein in actin severing is cofilin which enhances filament severing and breakdown by decorating actin filaments. Cofilin is activated by LIMK mediated phosphorylation.

Many PID patients harbor mutations in the genes encoding key proteins of the actin polymerization cascade (139, 140). As reorganization of the actin cytoskeleton is indispensable for IS formation, maturation, stability, and function (11), such mutations can have profound effects on the IS. Although formins, that directly nucleates actin polymerization, are required for various functional aspects of the IS (141–143), mutations in formin encoding genes have not yet been identified in PID patients. In contrast, mutations in regulators of actin polymerization mediated by the Arp2/3 complex are numerous. Of these, mutations in the WAS gene encoding WASp have been extensively studied and revealed the importance for actin cytoskeleton dynamics in tumor surveillance by cytotoxic cells. In the steady state, WASp is maintained in an inactive conformation that is stabilized by WIP (144). In addition, WIP acts as a chaperon by protecting WASp from both proteasome and calpain-mediated degradation. Upon intracellular transmission of activating signals, the active form of the Rho GTPase CDC42 (GTP-bound) binds to the GTPase binding domain of WASp. This interaction with CDC42 converts WASp into an active open confirmation that delivers actin monomers to the Arp2/3 complex. Consequently, the Arp2/3 complex initiates the formation of a branched actin network underneath the membrane of the IS (**Figure 5**).

#### Defects Associated With WASp

In PID patients deficient of WASp expression, this branched actin network is defective. As WASp contains multiple functional domains, the effects of the mutations on WASp can vary greatly depending on where the mutations are located on the WAS gene. Some WASp mutations result in half size truncated proteins that are devoid of the verprolin-cofilin-acidic (VCA) C-terminal domain. Without this VCA domain, the interaction with Arp2/3 is completely abolished (145) (**Figure 5**). Other mutations, in particular those in the proline rich region (PRR), can prevent WASp localization at the T cell:APC contact site. As a result, WASp has a diffuse pattern inside T cells and fails to polymerize actin at the IS (146, 147). A possible explanation for this mislocalization could come from the inability of WASp to interact with the adaptor proline-serine-threonine phosphatase interacting protein 1 (PSTPIP1) (148). Interestingly, a similar role for PSTPIP1 in IS formation has been described in CD4^+^ T cells from common CVID patients (149), suggesting that the TCR-PSTPIP1-WASp pathway can be altered in different PID. At the membrane level, the precise recruitment pathway of WASp is still controversial. While WASp is recruited within lipid rafts by Zap70 and CrKL (150), this is not the case for its PSTPIP1 mediated recruitment. It is interesting to note that CrKL is also one of the genes deleted in the DiGeorge syndrome, a PID associated with reduced NK cell cytotoxicity (151) and T cell proliferation (152). In addition, Zap70 activity is also required for CDC42 recruitment at the T cell:APC IS (147). These results indicate that Zap70 positioning at the membrane is critical for IS formation. In fact, membrane recruitment of Zap70 is dependent on the RhoH GTPase (153). Although the tyrosine kinase SYK can partially compensate for Zap70 loss in CD8^+^ T cells (154), both Zap70 and RhoH deficiencies lead to PID with alterations in T cells (155). WASp deficiency can also be caused by an absence of WIP. Without WIP, WASp is rapidly degraded. As a result, CD8^+^ T cells from WIP deficient patients cannot assemble an IS and thus perform cytolytic functions (156).

DCs and CD4^+^ T cells, both of which are required to support an optimal CD8^+^ T cell response, can also be affected by WASp deficiency. Indeed unstable synapses have been observed in murine WASp deficient DCs (157). Due to a shorter cell-cell interaction time, these DCs failed to prime T cells. Moreover, WASp deficient DCs migrate less efficiently from the periphery to secondary lymphoid organs, a major hurdle for tumor antigen presentation to CD8^+^ T cells (158). Interestingly, in the absence of WASp, CD8^-^ DCs acquire antigen cross-presentation ability that is dependent on Rac2, a Rho GTPase mutated in some PID (159, 160). In CD4^+^ T cells, the absence of WASp leads to unstable synapses and CD4^+^ T cells are then unable to uphold a sustained calcium flux required for their full activation (161). In addition, studies performed with murine CD4^+^ T cells have shown that the Fyn kinase needs to be recruited to the IS to activate WASp (148). Surprisingly, this type of WASp activation occurs independently of CDC42 binding.

Impaired NK cells lytic functions are also observed in WAS patients and WASp deficient mice (85, 86, 106, 107). In the absence of WASp, the NK cell IS is immature and lacks multiple signaling molecules. The local recruitment of proteins involved in calcium signaling, like Zap70 and calcineurin, is delayed (162). Consequently, the calcium-dependent nuclear translocation of the NFAT2 and NF-κB (RelA) transcription factors is reduced. Although their exact functions in NK cells are still unclear, their roles in cytokine gene transcription during T cell activation have been well characterized (163, 164). However, the lytic functions of WASp deficient NK cells can be restored by exposure to interleukin-2 (IL-2). This effect is mediated by WAVE2 and is thus completely independent of WASp (87). This result showed that IL-2 can trigger a WAVE2-dependent alternative actin reorganization pathway. In a similar manner, IL-2 can restore actin polymerization, degranulation and interferon-γ (IFN-γ) production in murine WASp KO NK cells (86). Tumors naturally producing IL-2 could thus be kept in check by murine WASp KO NK cells *in vivo*. Interestingly, the same study reported that high expression of WASp and IL-2 correlated with a better survival in neuroblastoma patients. All these different results paved the road for IL-2-based clinical trials in WAS patients (165). Other types of therapies, such as hematopoietic stem cells gene transfer, have been proposed to overcome WASp deficiency (166). In some rare cases, secondary mutations in the WAS gene can correct the DNA sequence modification caused by the first mutation and thus restore the submembrane localization of WASp (167).

#### Defects Associated With the Arp2/3 Complex

In addition to WASp mutations, mutations in the genes encoding for the different subunits of the Arp2/3 complex can also affect the IS stability and function. CD8^+^ T cells from patients lacking ARPC1B, a component of the Arp2/3 complex, fail to remain firmly attached to cancer cells, resulting in lower degranulation and killing efficiency (168). Additionally, these CD8^+^ T cells harbor defective proliferation due to reduced cell surface expression of the glucose transporter GLUT1, the CD8 co-receptor and the TCR. Interestingly, the microtubule cytoskeleton is unaltered in ARPC1B PID patients. Reduced IS area and conjugates number were reported in additional ARPC1B deficient patients by Brigida et al. (169). As the Arp2/3 complex needs actin monomers to induce branched polymerization, maintaining an intracellular actin monomer pool is essential. Accordingly, inhibition of the actin filament depolymerization can indirectly perturb Arp2/3 activity. This could explain why T cells from patients deficient for WDR1, a protein implicated in actin filament turnover, show impaired calcium influx and proliferation upon TCR engagement (156).

#### Defects Associated With LFA-1

The LFA-1 integrin present on the cell surface of cytotoxic lymphocytes is essential to ensure a tight adhesion to cancer cells. LFA-1 can adopt multiple conformations, which all differ in their ligand-binding affinities. Activation of LFA-1 and subsequent switch to its high affinity conformation are mediated by inside-out signaling (170). Defects in the inside-out signaling molecules, or mislocalization of LFA-1 and its partners at the plasma membrane can severely impair the IS. As all these steps are dependent on actin dynamics, any perturbation of the actin cytoskeleton can have harmful consequences for LFA-1 activation (171). When LFA-1 density at the IS is reduced, the conjugation frequency is decreased. DOCK8 is required for LFA-1 clustering at the NK cell IS (172). Low conjugation efficiency along with diminished cell cytotoxicity were found in NK cells from DOCK8 deficient patients (173). In contrast to WASp deficient NK cells, impaired IS formation of DOCK8 deficient NK cells could not be rescued by IL-2 treatment. This suggests that DOCK8 acts upstream of WASp and WAVE2 in activation-induced actin cytoskeleton reorganization. As for NK cells, CD8^+^ T cells are affected by DOCK8 deficiency indicated by absence of LFA-1 clustering at the T:DC synapse using murine cells (174). This incorrect priming leads to decreased proliferation and survival of DOCK8 deficient CD8^+^ T cells. The T:DC synapse stability is also impaired in the absence of the CXCR4 chemokine receptor, as observed in WHIM PID patients (175). This could be due to the ability of CXCR4/CXCL12 axis to control LFA-1 conformation, a process known as chemokine induced inside-out signaling (176). Interestingly, T cells from PID patients deficient for moesin have an overexpression of LFA-1 but no alteration in IS formation was reported (73). However, as the proliferation of moesin deficient T cells was decreased, the signal transduction function of the IS may still be defective. Signals coming from the TCR or chemokine receptors transit through integrin binding adaptor proteins to modify the affinity of LFA-1. Such adaptors, like talin and kindlin, must be recruited to the IS to ensure LFA-1 activation (177). T cells from LAD3 syndrome patients, who are deficient for kindlin-3, fail to spread on DCs upon TCR stimulation (178) or on ICAM-1-coated slides (179). In regard to talin, its recruitment at the IS depends on the cell type. Indeed, while this process is WAVE2-dependent in T cells (180), it is mediated by DOCK8 in NK cells (172). This study also reported that DOCK8 mediates WASp recruitment. These results suggest that WASp can be recruited at the IS by various adaptors proteins. Finally, the kinase MST1 has been shown to induce the activation of DOCK8, notably upon chemokine stimulation (181, 182). In addition, MST1 and the downstream kinase NDR1 are required for kindlin-3 recruitment at the T cell IS (183). Defective LFA-1/ICAM-1-dependent cellular adhesion has been described in PID patients deficient for MST1 (184).

### PID Affecting the Secretion Cytotoxic Granules

Although an intact actin cytoskeleton is mandatory for the formation of a stable IS, it is not sufficient to ensure cancer cell killing. Upon formation of a stable synapse, the second part of the target cell lysis process, when cytotoxic granules migrate towards the IS and release their content, can be affected in PID patients too. The secretion of cytotoxic granules from NK cells and CD8^+^ T cells requires the coordination of both microtubule and actin cytoskeletons with different vesicular pathways (185). All these steps involve multiple membrane fusions events, mostly driven by the Rab, SNARE and Munc proteins families (186–188). In addition to the mutations affecting the microtubule and the actin cytoskeletons, genetic alteration in these protein families can also result in impaired secretion of cytotoxic molecules into the synaptic cleft. Such defects are at the basis of various PID.

#### Polarization of Lytic Granules to the IS

**Figure 6:**
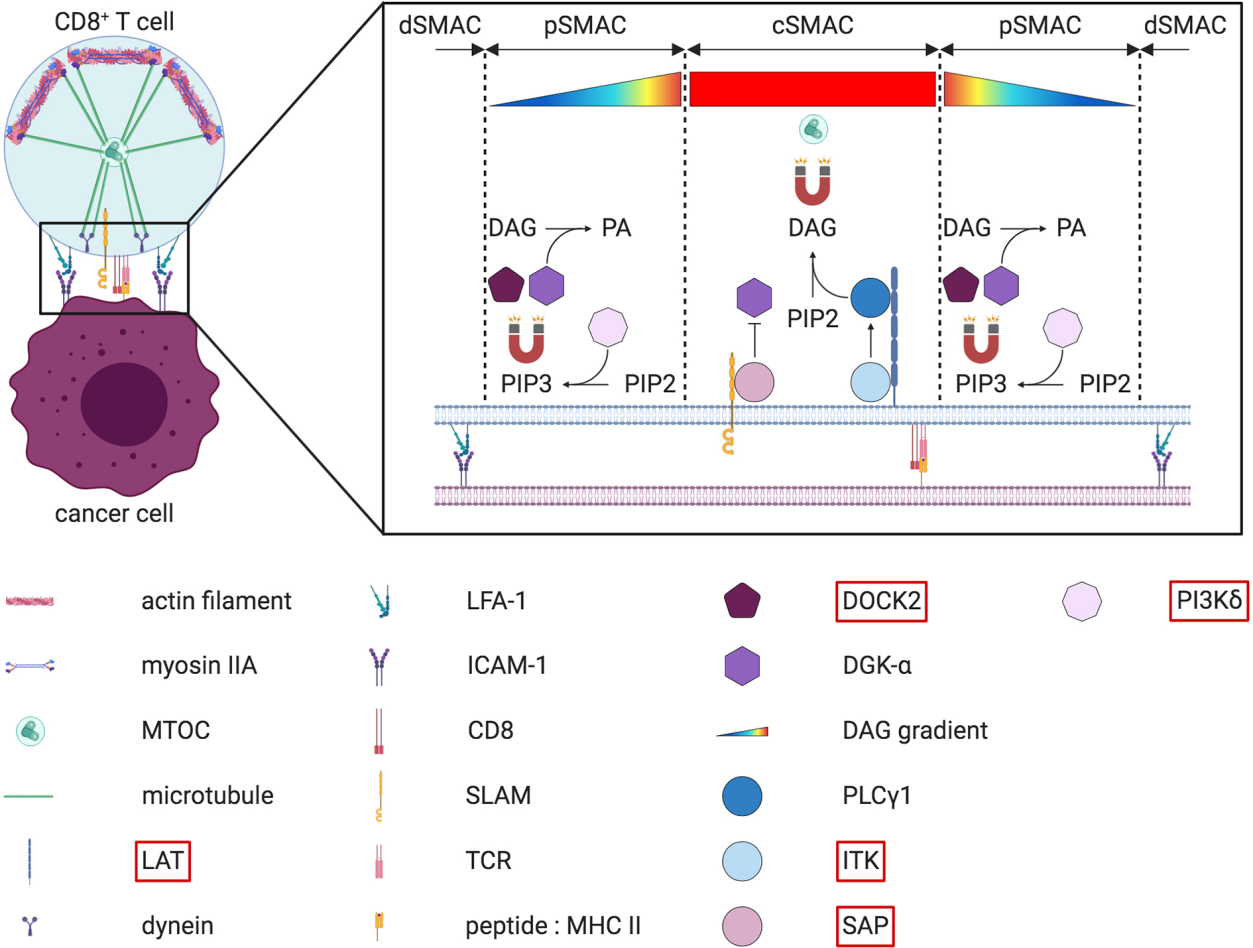
“Push and pull” model for MTOC polarization at the IS and DAG gradient at the cSMAC in CD8^+^ T cells. On the left part of the figure, the MTOC is relocated at the IS thanks the joint effort of both dynein and myosin IIA. Upon actin depletion at the cSMAC, dynein is anchored at the synaptic membrane from where it “pulls” on the microtubules to reposition the MTOC closer to the IS. At the same time, myosin IIA “pushes” microtubules from the opposite cell side. The right part of the figure depicts the diacylglycerol (DAG) gradient present at the IS. This gradient, which increases from the outside to the inside of the IS, guides the microtubule-driven MTOC reorientation at the IS. Several key enzymes needed to generate this DAG gradient are described more in detail in the text. Note that a simplified version of the LAT signalosome is depicted. The molecules highlighted in red have been found mutated in some PID.

**Figure 7:**
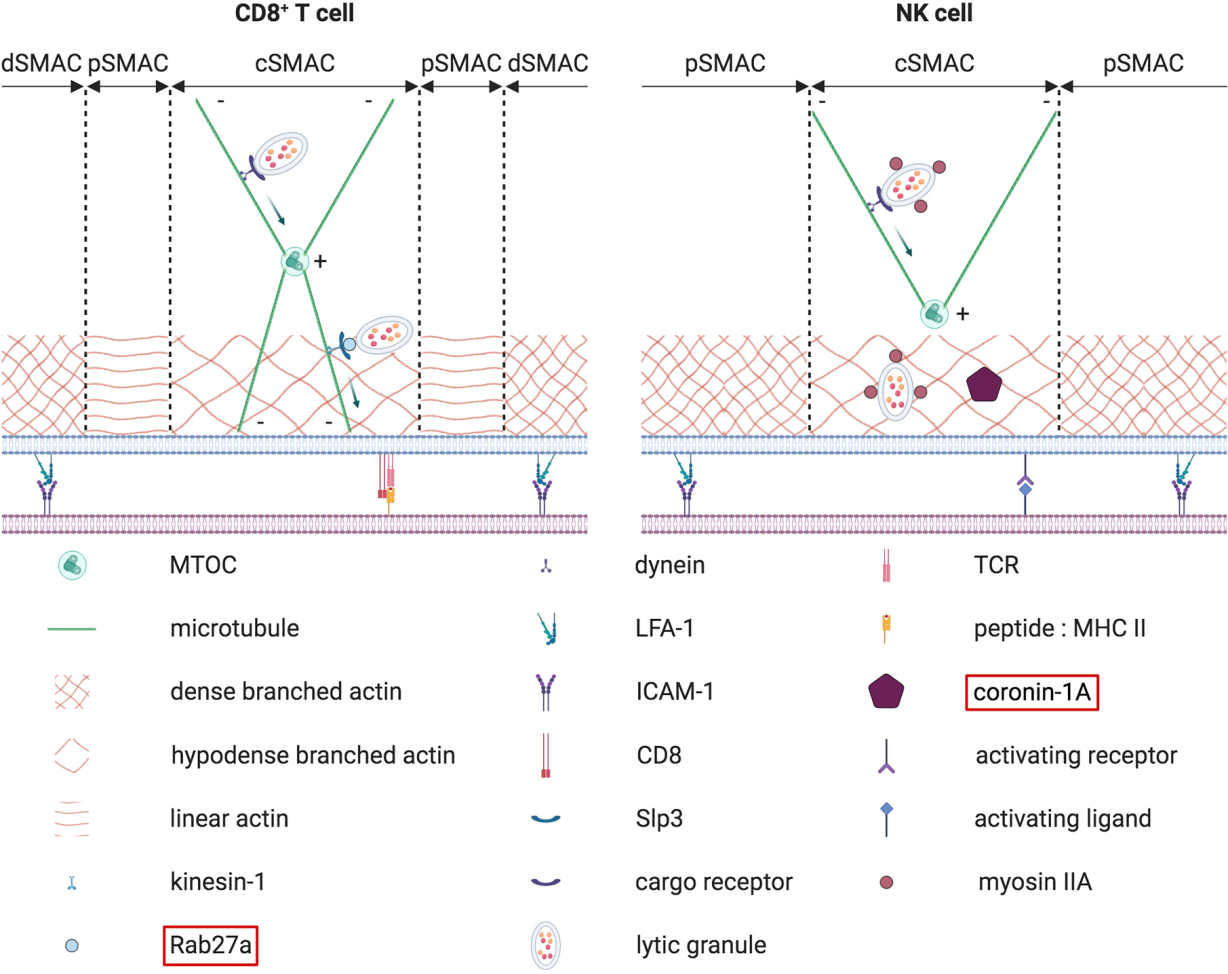
Terminal transport of lytic granules from the polarized MTOC to the synaptic membrane. The terminal transport of lytic granules from the polarized MTOC to the synaptic membrane differs between CD8^+^ T cells (on the left) and NK cells (on the right). In CD8^+^ T cells, lytic granules first converge to the MTOC in a retrograde dynein-dependent process. Then an anterograde transport mediated by Rab27a, kinesin-1 and Slp3 allows lytic granules to cover the small distance separating the polarized MTOC from the membrane. In NK cells, such an anterograde final transport has not been described so far. Instead, lytic granules are first associated with myosin IIA. Myosin IIA later helps lytic granules to navigate through the synaptic actin network by interacting with F-actin. Concomitantly, coronin 1A is required to reduce the density of the actin mesh, thereby facilitating lytic granules approach to the membrane. The molecules highlighted in red have been found mutated in some PID.

Before their secretion at the contact site, cytotoxic granules first need to reach and accumulate at the IS. Upon target recognition, lytic granules rapidly cluster around the microtubule-organizing center (MTOC). Convergence of these cytotoxic granules towards the MTOC is dependent on dynein and microtubules (189, 190). The MTOC with adjacent lytic granules migrates towards to the IS. In T cells, the MTOC repositioning at the IS is mediated by a diacylglycerol (DAG) gradient (191) (**Figure 6**). This gradient of DAG must be tightly regulated, with a perfect balance between DAG synthesis and DAG degradation. Multiple enzymes are required to shape this gradient, and several of them have been found mutated in different PID. DAG is a secondary lipid messenger that is generated upon hydrolysis of phosphatidylinositol 4,5 bisphosphate (PIP2) by the phospholipase Cγ1 (PLCγ1). PLCγ1 is part of a multiprotein complex that becomes activated by the interleukin-2-inducible T cell kinase (ITK) upon TCR signaling (192). Mutations affecting the phosphorylation and activation of ITK lead to a PID with impaired degranulation of CD8^+^ T cells (193, 194). Thus, orchestration of this cascade of enzymes and kinases is essential for DAG synthesis. To maintain optimal DAG levels, a fine-tuning of the enzyme responsible for DAG degradation, the diacylglycerol kinase-α (DGK-α), is important. To generate a DAG gradient with increasing concentration from the periphery towards the center of the IS, DGK-α is specifically recruited to the pSMAC (195). In the pSMAC, synaptic PIP3 is directly responsible for both the recruitment and the activation of DGK-α. It is produced upon phosphorylation of PIP2 by the phosphoinositide 3-kinases (PI3K), especially PI3Kδ. Interestingly, mutations in the subunits of PI3K also translate into multiple PID associated with decreased NK cell cytotoxicity (196, 197) and CD8^+^ T cell proliferation (198). In addition to DGK-α, PIP3 also recruits DOCK2, which is needed to drive a Rac1/WAVE2-dependent actin polymerization at the pSMAC (199, 200). DOCK2 deficiency has been shown to negatively alter both NK cell and CD8^+^ T cell degranulation and is recognized as a PID (199, 201). At the same time as the peripheral recruitment of DGK-α, a supplementary safeguard mechanism also ensures DGK-α inhibition at the cSMAC. This inhibitory signal is transmitted by the SAP adaptor protein (202). In the absence of functional SAP, as observed in XLP-1 patients, the killing of EBV-infected B cells by NK cells is severely compromised (203–205). Additional data obtained from murine cells suggest that MTOC polarization and cytotoxicity can also be impaired in SAP-deficient CD8^+^ T cells (206). All these results demonstrate that the membrane local pool of PIP2 plays a key role in the establishment of the DAG gradient. As a consequence, this PIP2 pool is constantly replenished by the phosphatidylinositol 4-phosphate 5-kinases (PIP5K) (207, 208). In T cells, once the DAG gradient is established, actin filaments can be cleared from the cSMAC, allowing the microtubule related motor protein dynein to be recruited (209). This depletion of cortical actin is critical for the MTOC repositioning at the IS and subsequent lytic granules secretion (210, 211). These studies emphasize the involvement of the actin cytoskeleton for the MTOC polarization (141). Alterations in actin polymerization can also negatively impact the MTOC and lytic granules polarization at the IS. Simultaneously to dynein accumulation at the IS, the actin-based myosin IIA is relocalized at the opposite cell side of the IS (212). Together, dynein and myosin IIA generate the mechanical forces necessary to recruit the MTOC in a “push and pull” manner (212) (**Figure 6**). In NK cells, an additional role of myosin IIA in the progression of lytic granules through the synaptic actin network has been suggested (213, 214) (**Figure 7**).

#### Exocytosis of Polarized Lytic Granules

**Figure 8:**
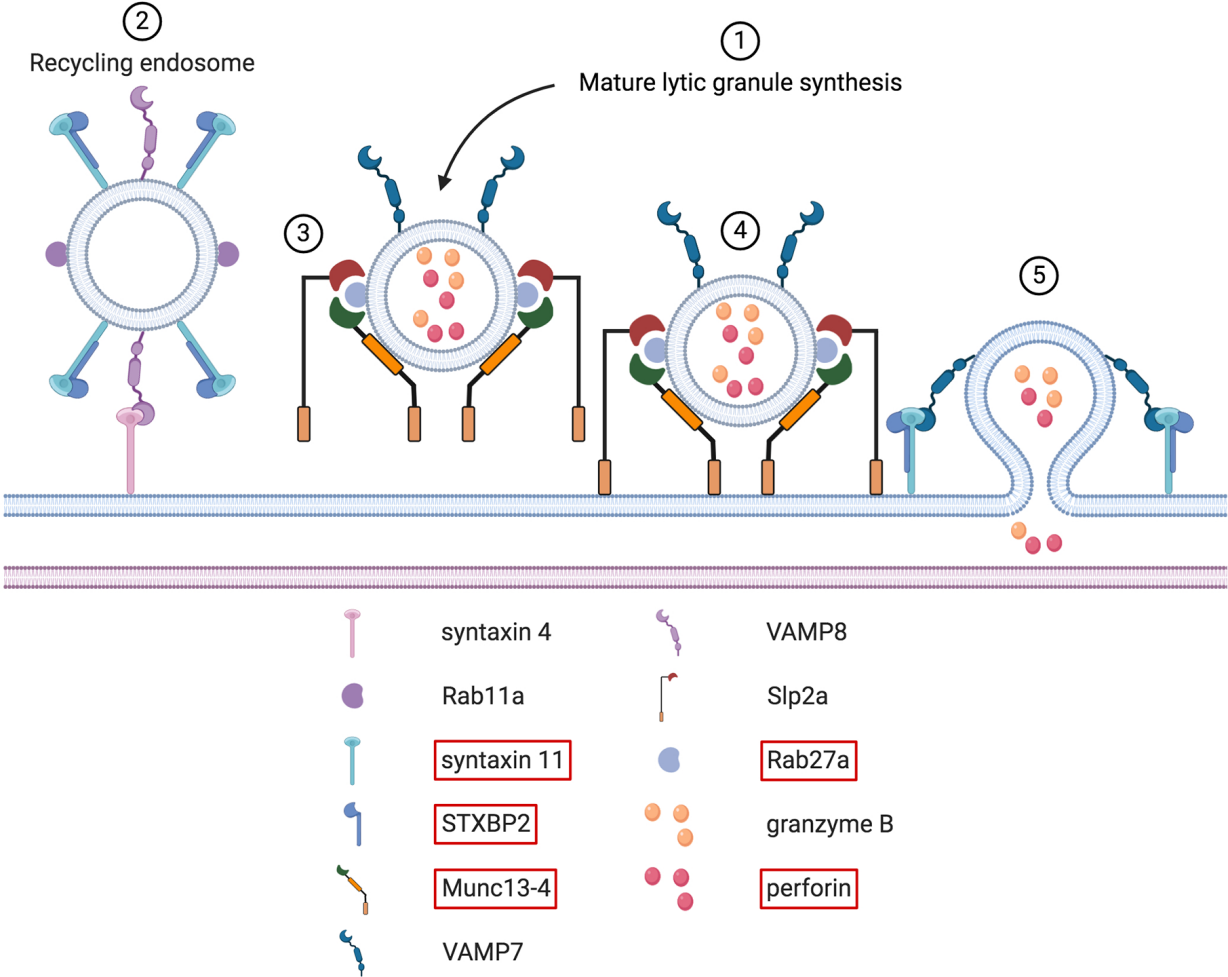
Docking, tethering, priming and fusion of lytic granules at the IS. Upon immune cell activation, synthesis of mature lytic granules occurs (step # 1). Several molecules, including LYST and the AP-3 complex, are involved in this process (not shown here). These two proteins are causative in the Chediak-Higashi syndrome and the type 2 Hermansky-Pudlak syndrome, respectively. Once mature lytic granules have reached the IS, sequential steps must take place to guarantee their proper docking, tethering, priming and fusion with the synaptic membrane. Before the arrival of lytic granules, Rab11a^+^ recycling endosomes fuse with the plasma membrane through a VAMP8-syntaxin 4 interaction (step # 2). Upon this fusion, syntaxin 11/STXBP2 complexes are deposited at the plasma membrane. After newly arrived lytic granules are docked at the IS (step # 3), tethering factors ensure that these granules remain firmly in place. This is mediated by several tethering proteins, such as Munc13-4 and Slp2a (step # 4). Simultaneously, Munc13-4 primes the lytic granules for the final fusion stage. Syntaxin 11/STXBP2 complexes, formerly brought by recycling endosomes, mediate the fusion of lytic granules with the synaptic membrane by interacting with VAMP7. After this fusion step, cytotoxic molecules present within the lytic granules are released within the synaptic cleft (step # 5). Note that the interfacial actin protrusions described in CD8^+^ T cells by Tamzalit et al. (215) are not shown.

Once the cytotoxic granules have reached the IS, several sequential steps must occur to allow the release of their content within the synaptic cleft (**Figure 8**). Different PID are characterized by alterations of these steps. Among them are the different forms of familial hemophagocytic lymphohistiocytosis (FLH). HL is characterized by an uncontrolled activation of cytotoxic lymphocytes, which results in the hypersecretion of inflammatory cytokines. This cytokine storm syndrome often leads to fatal multi-organ failure. Depending on the gene affected, different FHL subtypes can be defined (216). As the secretory pathway is similar between NK cells and CD8^+^ T cells, both cell types are equally affected in FHL (217).

##### Type 1 and Type 2 FHL

Although the gene mutated in type 1 FHL is located on chromosome 9, its exact name is currently unknown. Type 2 FHL is caused by mutations in the gene encoding for perforin (218). In these patients the majority of mutations lead to loss of perforin expression. As a result, the killing capacity of their cytotoxic lymphocytes is severely impaired. As the exocytosis machinery of these patients is normal, this subtype will not be further discussed within the scope of this special research topic (219–221).

##### Type 3 FHL

Type 3 FHL is linked to mutations in the UNC13D gene. This gene encodes the Munc13-4 protein that is implicated in the cytotoxic granules priming phase, the last step before fusion with the plasma membrane (222) (**Figure 8**). Consequently, the earlier steps of IS formation, including the microtubule mediated movement of granules towards the IS, are not affected. Data obtained with murine NK cells have shown that calcium binding of Munc13-4 is essential for degranulation (223). This could potentially explain why PID affecting calcium channels also result in NK cells and CD8^+^ T cells degranulation defects (224). Interestingly, studies performed on murine CD8^+^ T cells suggest that the remaining degranulation observed in the absence of Munc13-4 could be due to the expression of the Munc13-1 isoform (225). Upon NK cell activation, Munc13-4 is recruited in membrane lipid rafts in a PIP2-dependent process (226). PIP2 controls Munc13-4 recycling that is essential to ensure serial killing, a process where a cytotoxic cell can kill multiple target cells sequentially. These additional pieces of data further highlight the importance of PIP2. Regarding possible treatments options, IL-2 therapy is unfortunately not conceivable for FHL3 patients. However, they could potentially benefit from retroviral gene transfer therapy to re-establish CD8^+^ T cell cytotoxicity (227).

##### Type 4 FHL

Abnormalities in the syntaxin 11 protein are accountable for type 4 FHL (**Figure 8**). Although different types of defects have been reported, they all lead to an impaired degranulation of both NK cells and CD8^+^ T cells (228). Some of the type 4 FHL can alter the conformation of syntaxin 11 while preserving its activity (229). This residual activity observed in NK cells could explain why some patient show signs of type 4 FHL only later in their life. Other mutations can affect the recruitment of syntaxin 11 at the IS. Transfection of different syntaxin 11 mutant constructs in the YTS NK cell line revealed that the C-terminal domain is essential for its correct localization. Without this carboxy-terminal region, syntaxin 11 is no longer S-acylated and remains dispersed in the cytoplasm (230). Initial studies suggested that CD-M6PR^+^ late endosomes were responsible for syntaxin 11 deposit at the NK IS (231). However, more recent data obtained from CD8^+^ T cells suggest that syntaxin 11 rather is localized within Rab11a^+^ recycling endosomes (232) (**Figure 8**). This specialized endosome subtype is necessary to convey syntaxin 11 at the IS prior the arrival of lytic granules, without which they fail to dock at the membrane. The interaction between VAMP8 and syntaxin 4 is required for the fusion of these recycling endosomes with the plasma membrane (233, 234) (**Figure 8**). Upon fusion, syntaxin 11 is transferred to the plasma membrane and can interact with VAMP7 that is present on arriving cytotoxic granules (235) (**Figure 8**). Alterations in VAMP7, VAMP8, and syntaxin 4 can lead to impaired degranulation and killing. In addition, defects in VAMP7 could also result in defective CD8^+^ T cell activation, provided that the VAMP7 mediated recruitment of LAT at the IS is conserved between T cell subsets (236, 237). At the end, NK cell degranulation can also be partially restored upon IL-2 treatment (238).

##### Type 5 FHL

Type 5 FHL is characterized by mutations in the STXBP2 gene, encoding STXBP2 (also known as Munc18-2) (239, 240) (**Figure 8**). These two studies were the first to show a direct interaction between STXBP2 and syntaxin 11 (**Figure 8**). In addition, several FHL5 patients had a decreased expression of syntaxin, suggesting that STXBP2 acts as a chaperon for syntaxin 11. For this interaction, both the N terminal part and the Habc domain of syntaxin 11 must be intact (230, 241). However, STXBP2 alone is not sufficient to guarantee syntaxin 11 expression. Proteasome-mediated caspase degradation is also required (231). Moreover, syntaxin 11 cannot be transferred to the plasma membrane in the absence of STXBP2 (242). At the IS, STXBP2 promotes the assembly of syntaxin 11 containing SNARE complexes (233, 243). This step is essential to ensure complete membrane fusion. Similar to FHL4, degranulation can be partially restored with IL-2 (244). It has been suggested that STXBP1 could potentially drive this IL-2-dependent rescue pathway in the absence of STXBP2 (245).

##### Type 2 Griscelli Syndrome

Contrary to FHL where HL is the sole clinical manifestation, HL can also develop occasionally in patients initially affected by others PID (246). One of these PID is the type 2 Griscelli syndrome (GS2). GS2 is caused by mutations in RAB27A (247) (**Figure 8**). This gene encodes for the Rab27a Ras-like GTPase. Initial studies using *ashen* mice (deficient for Rab27a) showed that Rab27a is involved in the docking of lytic granules at the membrane in both NK and CD8^+^ T cells but not in their microtubule dependent polarization (248, 249). This was later confirmed in human CD8^+^ T cells deficient for Rab27a (222). Rab27a is not only required for the docking but also for the tethering of lytic granules at the membrane (250) (**Figure 8**). Interestingly, this tethering function of Rab27a is dependent on its interaction with Munc13-4, the protein mutated in FHL3. In addition to Munc13-4, Rab27a interacts with Slp2a to enhance the tethering (185, 251). Mutations disrupting the binding with Rab27a interacting proteins, including Munc13-4, are found in GS2 patients (252–254). Moreover, the interaction between Rab27a and Munc-13-4 could be necessary to coordinate the transition between the tethering and priming steps (185, 255). A detailed description of the Rab27a-Munc13-4 interaction has recently been published (187). Finally, in CD8^+^ T cells, Rab27a is implicated in the anterograde transport of lytic granules from the polarized MTOC to the plasma membrane (256, 257) (**Figure 7**).

##### Chediak-Higashi Syndrome

The Chediak-Higashi syndrome (CHS) is a PID with clinical manifestations similar to those observed in GS2 patients. The hallmark of CHS is the presence of giant lysosomes-related organelles, including cytotoxic granules, inside the cells. This is due to mutations in the gene encoding the lysosomal trafficking regulator LYST. About 85% of CHS patients progress into an accelerated phase which is identical to HLH (258). As seen in the other forms of HLH, both CD8^+^ T cells and NK cells from CHS patients have an impaired degranulation (259–261). LYST contains several functional domains that do not result in the same pathophysiological effects when mutated. Mutations in the BEACH domain lead to the production of a normal number of slightly enlarged lytic granules in NK cells. However, these granules are not able to polarize to the IS and thus remain scattered in the cytoplasm. The reason for this defect in polarization remains unclear. On the other hand, mutations in the ARM/HEAT domain induce the formation of a small number of large lytic granules. Although these granules can migrate to the IS, they cannot efficiently be exocytosed, suggesting that the size of the actin meshwork at the cSMAC could block their passage (262), a hypothesis proposed based on work from the Orange and Davis’ labs (263, 264). In 2018, Gil-Krzewska et al. confirmed it by using LYST-deficient NK-92 MI cells (265). Even though the machinery required for granule polarization, docking, priming and fusion of lytic granules are unaffected, these cells were unable to degranulate. Reduction of the actin network density or restoration of the normal size of lytic granules through Rab14 silencing corrected the degranulation defect, showing that the enlarged lytic granules were too big to navigate through the cortical actin mesh. Interestingly, it appears that this lytic granule size defect is more prominent in NK cells than in CD8^+^ T cells (266). This defect is highly reminiscent of the one observed in patients deficient for coronin-1A (267). In these patients the reverse situation occurs: the lytic granules have a normal size, but the cortical actin network is too dense and thus not permissive enough for lytic granules to move properly (**Figure 7**).

##### Type 2 Hermansky-Pudlak Syndrome

HLH is also one of the complications of patients affected by the type 2 Hermansky-Pudlak syndrome (HPS2) (268, 269). HPS2 is caused by mutations in AP3B1, the gene encoding the β3A subunit of the AP-3 complex (270). AP-3 is a multimeric complex involved in the organelle synthesis and early endosomal protein sorting. When the conformation of the β3A subunit is altered, the whole AP-3 complex becomes unstable and susceptible to degradation (271). In the absence of AP-3, the cytotoxic potential of CD8^+^ T cells is severely reduced. This is due to the inability of lytic granules to move along microtubules towards the MTOC and thus to polarize at the IS (272). Interestingly, this study also reported that the lytic granules were enlarged, a phenotype reminiscent of CHS. Moreover, the degranulation of NK cells is impaired (273). Occasionally, some mutations located close to the C-terminal end only result in a mild HPS2, where the NK cell degranulation is still impaired but the NK cells and CD8^+^ T cells cytotoxic response is normal (274). In addition to the β3A subunit, problems with NK cells and CD8^+^ T cells degranulation are observed when the δ subunit is mutated (275). Given that this subunit is common to both the ubiquitous and the neuronal AP-3 forms, these patients also experienced neurologic disorders. The authors proposed to name this new PID HPS10 to distinguish it from HPS2.

## Concluding Remarks

Over the last decade, the positive correlation between PID and cancer has become more obvious. This observation is further supported by a growing body of literature and has been the subject of a special issue in Frontiers In Immunology in 2019 (276). Several cancer risk factors have been clearly identified in PID patients. Recurrent bacterial, viruses and fungal infections are some of the major factors. Chronic inflammation triggered by persistence of *H. pylori* is often preceding gastric cancer appearance (277–279), a phenomenon drastically worsened in some PID patients (280, 281). Similar observations were made for fungal infections (282) or for the Epstein Barr Virus, as recently highlighted in another special issue of Frontiers In Immunology (283). Due to their impaired immune system, PID patients fail to efficiently eradicate these cancer promoting pathogens. The immunocompromised environment of PID patients fails to successfully stop the proliferation of cancer promoting pathogens and is incapable of efficiently eliminating tumor cells once they appear. In PID patients, the tumor “search and destroy” process orchestrated by cytotoxic CD8^+^ T cells and NK cells is often defective. A lack of both CD8^+^ T cells and NK cells, as described in certain severe combined immunodeficiencies, can easily render tumor immunosurveillance almost non-existent. However, some alterations commonly found in PID patients are subtler in the sense that they impair the functions of cytotoxic lymphocytes rather than simply preventing their development. This is notably the case for genetic mutations with negative consequences on the formation, stability or the function of the IS.

In addition to NK cells and CD8^+^ T cells (including the accessory DCs and CD4^+^ helper T cells required for the CD8^+^ T cell response), other immune cell types can assemble a synapse. Dysfunction in these types of synapses can contribute directly or indirectly to cancer onset and progression. T regulatory (Tregs) cells, a subset of CD4^+^ T cells, is one of these cell types. In the absence of WASp, Tregs are unable to stop the proliferation of B cells (284). However, they can control the proliferation of conventional T (T conv) cells. This study provides one possible explanation why B cell malignancies are more frequently observed among WAS patients (99). Nevertheless, the reason why the Tregs : Tconv synapse is still functional remains unclear and warrants further investigations. Besides Tregs, phagocytic cells can also form a synapse. This synapse, which is called a phagocytic synapse, shares many features with the IS, including prominent remodeling of the actin cytoskeleton (285). It is thus not surprising that the phagocytic activity by macrophages is impaired in the absence of WASp (286–288). This point is particularly interesting because multiple studies showed that macrophages are also important players in tumor immunosurveillance (289). Given that CD8^+^ T cells and NK cells draw most of the attention, the specific role of macrophages in tumor cell eradication in the context of PID remains to be explored. Abnormalities in neutrophil mediated phagocytosis may be associated to cancer progression too. Indeed, activated neutrophils from patients affected by chronic granulomatous disease (CGD) are unable to kill non-opsonized K562 cancer cells *in vitro*, a defect linked to lower reactive oxygen species (ROS) production (290). However, elimination of antibody-coated K562 cancer cells was unaffected, suggesting a ROS-independent mechanism in this specific situation. Mechanical disruption of the cancer cell membrane has recently been proposed as the mechanism behind this ROS-independent cancer cell death (291). This new killing mechanism, named trogocytosis, requires a cell-cell contact that is dependent on the integrin Mac-1 and might also involve an actin-myosin contraction phase. Accordingly, it is highly conceivable that actin cytoskeleton alterations, such as those described here, could inhibit this process. A careful analysis of this newly described mechanism and its potential impairment in the pathogenesis of PID related cancers needs to be conducted.

Some genetic mutations found in PID patients can pose major hurdles to some anticancer therapies, such as CAR-T cells. CAR-T cell is an adoptive cell transfer therapy that has been approved as a standard of care for some lymphomas and leukemia, two of the most frequent tumor types found in PID patients (292). However, autologous CAR-T cells cannot be considered as a curative cancer treatment in PID patients. Indeed, as the transfused CAR-T cells are derived from T cells isolated from the patient’s own blood, they still carry the same genetic defect (292). Given that CAR-T cells need to form an IS with cancer target cells, the treatment is likely to fail. However, it should be noted that as the CAR-T cell synapse is less dependent on LFA-1 and shows a smaller actin ring, CAR-T cells from PID patients may be functional despite defects in actin polymerization (293). But with a cumbersome and costly process associated to an unpredictable outcome, autologous CAR-T cells are not a realistic option for PID patients (294). An alternative would be to use allogeneic CAR-T cells (295). As the infused T cells are derived from an unrelated donor, the genetic mutation initially found in the patient is no longer present. However, such adoptive transfer can lead to life-threatening complications such as the graft-versus-host disease (GVHD). “Off-the-shelf” CAR-NK cells, a promising therapy able to overcome GVDH while maintaining production costs low, could be more appropriate (296, 297).

Like many other diseases, the best strategy is to adopt a preventive approach by directly correcting the genetic defect itself. This greatly reduces the risk of developing cancer. Therefore, among all the treatments options currently available for PID patients, hematopoietic stem cell transplantation (HSCT) is the most curative and reliable one. Multiple studies have reported significant efficacy of HSCT in different PID associated with synaptic defects (92, 201, 298–305). However, HSCT is a heavy surgical procedure that requires prior immunosuppressive conditioning. Determining the best intensity of such regimens is essential before injection in individuals with already an impaired immune system (306). In addition, several complications can arise after HSCT (307, 308). Despite the recent progress achieved in HSCT, the benefit/risk ratio must be carefully assessed for each patient (309). The fact that some of these PID can be managed without HSCT makes this assessment even more critical (310). Alternative strategies are emerging and could potentially replace HSCT for specific PID (165). Until such treatments become approved as first line therapies, regular monitoring of PID patients associated with new routine procedures is critical to potentially detect early signs of cancer (310), especially as NK cytotoxicity issues have recently been identified in other PID (311–313).

## Author Contributions

JM and MS wrote the manuscript and designed the figures. HW provided guidance to draft the plan of the manuscript. MK participated in the writing of the introduction. JM, MS, HW, LW, and CT edited the manuscript. All authors contributed to the article and approved the submitted version.

## Funding

This work was supported by a postdoctoral fellowship from Wenner-Gren foundations to MS, the Swedish Research Council, Cancer Society, Childhood Cancer Fund, a StratCan BlueSky award, the European Commission 7^th^ framework program Marie Curie reintegration grant (#249177), Åke Olsson foundation, Åke Wiberg Foundation, Bergvall Foundation, King Gustaf V’s 80-year Foundation, and Karolinska Institutet to LW. The work done by CT’s group is supported by Cancer Foundation Luxembourg (FC/2019/02), the National Research Fund (C19/BM/13579644 and PRIDE15/10675146/CANBIO), and Think Pink Lux.

## Conflict of Interest

The authors declare that the research was conducted in the absence of any commercial or financial relationships that could be construed as a potential conflict of interest.

## Abbreviations

A-EJ, alternative end joining; AID, activation-induced cytidine deaminase; APC, antigen presenting cell; Arp2/3, actin-related proteins 2/3; BCR, B cell receptor; BER, base excision repair; C, constant (segment in the IgH locus); CSH, Chediak-Higashi syndrome; CSR, class switch recombination; CVID, common variable immunodeficiency; DAG, diacylglycerol; DC, dendritic cell; DOCK, dedicator of cytokinesis; DSB, DNA double strand breaks; DZ, dark zone; EBV, Epstein-Barr virus; F-actin, filamentous actin; FDC, follicular dendritic cell; FHL, familial hemophagocytic lymphohistiocytosis; GC, germinal center; GS, Griscelli syndrome; HPS, Hermansky-Pudlak syndrome; HR, homologous recombination; Ig, immunoglobulin; IgH, immunoglobulin heavy chain; Igk, immunoglobulin kappa light chain; Igl, immunoglobulin lambda light chain; IgL, immunoglobulin light chain; IS, immunological synapse; ITK, inducible T cell kinase; LZ, light zone; MHC, major histocompatibility complex; MMR, mismatch repair; MTOC, microtubule-organizing center; N, non-germline-encoded nucleotides; NHEJ, non-homologous end joining; NK cell, natural killer cell; P, palindromic (nucleotides); PI3K, phosphoinositide 3-kinases; PID, primary immunodeficiency disorders; PIP2, phosphatidylinositol 4,5 bisphosphate; PLCγ1, phospholipase Cγ1; PPR, proline rich region; PSTPIP1, proline-serine-threonine phosphatase interacting protein 1; RAG, recombination activation gene; ROS, reactive oxygen species; RSS, recombination signature sequences; SHM, somatic hypermutation; SMAC, supra-molecular activation cluster.
